# Exploring the Impact of Noise and Image Quality on Deep Learning Performance in DXA Images

**DOI:** 10.3390/diagnostics14131328

**Published:** 2024-06-22

**Authors:** Dildar Hussain, Yeong Hyeon Gu

**Affiliations:** Department of Artificial Intelligence and Data Science, Sejong University, Seoul 05006, Republic of Korea; hussain.bangash@sejong.ac.kr

**Keywords:** dual-energy X-ray absorptiometry (DXA), osteoporosis, deep learning, segmentation, FCN, noise, imperfection, filters

## Abstract

Background and Objective: Segmentation of the femur in Dual-Energy X-ray (DXA) images poses challenges due to reduced contrast, noise, bone shape variations, and inconsistent X-ray beam penetration. In this study, we investigate the relationship between noise and certain deep learning (DL) techniques for semantic segmentation of the femur to enhance segmentation and bone mineral density (BMD) accuracy by incorporating noise reduction methods into DL models. Methods: Convolutional neural network (CNN)-based models were employed to segment femurs in DXA images and evaluate the effects of noise reduction filters on segmentation accuracy and their effect on BMD calculation. Various noise reduction techniques were integrated into DL-based models to enhance image quality before training. We assessed the performance of the fully convolutional neural network (FCNN) in comparison to noise reduction algorithms and manual segmentation methods. Results: Our study demonstrated that the FCNN outperformed noise reduction algorithms in enhancing segmentation accuracy and enabling precise calculation of BMD. The FCNN-based segmentation approach achieved a segmentation accuracy of 98.84% and a correlation coefficient of 0.9928 for BMD measurements, indicating its effectiveness in the clinical diagnosis of osteoporosis. Conclusions: In conclusion, integrating noise reduction techniques into DL-based models significantly improves femur segmentation accuracy in DXA images. The FCNN model, in particular, shows promising results in enhancing BMD calculation and clinical diagnosis of osteoporosis. These findings highlight the potential of DL techniques in addressing segmentation challenges and improving diagnostic accuracy in medical imaging.

## 1. Introduction

Osteoporosis is a pathological condition that compromises the integrity of the skeletal system. It serves as the primary factor for hip fractures within many countries. It demonstrates an absence of gender discrimination and has the potential to materialize at any point during an individual’s lifespan. A high proportion, exceeding 20%, of individuals affected with hip fractures succumb to the resultant trauma [1,2]. The disease can adequately be diagnosed with a low-dose X-ray imaging technique called dual-energy X-ray absorptiometry (DXA), which is considered a golden standard for diagnosing osteoporosis fracture risk [3]. Quantitative Computed Tomography (QCT) is another alternative. However, QCT requires a high dose of X-rays and is costly.

The initial step in the precise osteoporosis diagnosis involves accurate segmentation, which is crucial for accurately calculating bone mineral density (BMD) and final osteoporosis report generation. Segmentation errors can significantly impact the BMD calculation and subsequent analyses. However, several reasons drive incorrect segmentation in DXA images and post-analysis [4,5]. Firstly, the use of low-dose X-rays in DXA imaging provides noisy images. Secondly, there is an overlap of organs in human bodies. Thirdly, the irregular attenuation of the X-rays through the human body produces negative shadows, which appear as dark areas in the images. Other factors affecting segmentation quality include scanning orientation, luminocity intensities, resolution, and individual variations [6,7].

Noise profoundly impacts medical imaging analysis, manifesting as artifacts, reduced image clarity, and potential misinterpretation of diagnostic information. It stems from diverse sources like equipment limitations, patient motion, and inadequate radiation doses, distorting image features and complicating accurate identification and analysis of anatomical structures or pathologies. Moreover, noise undermines automated image processing algorithms, compromising segmentation, feature extraction, and classification tasks. Hence, effective noise reduction techniques are imperative for bolstering the accuracy and reliability of medical imaging analysis, thereby refining diagnostic interpretation and patient care [8,9].

In medical imaging, noise significantly disrupts segmentation processes by introducing inaccuracies and artifacts, hindering the precise delineation of anatomical structures. Obscured tissue boundaries due to noise often yield incomplete or erroneous segmentation results, while disrupted intensity gradients impede segmentation algorithms in distinguishing different regions of interest. Consequently, noise reduction techniques play a pivotal role in enhancing the accuracy and reliability of segmentation, ensuring meticulous delineation of structures, and maximizing the utility of imaging data for diagnostic and therapeutic endeavors [10,11,12].

Noise poses a substantial challenge to the performance of Deep Learning (DL) models in medical imaging by introducing uncertainties and inconsistencies in training data. Its presence obfuscates relevant features and patterns, leading to diminished model accuracy and reliability. Furthermore, noise exacerbates overfitting, wherein models learn to capture noise instead of meaningful information, impairing generalization to new data. Robust preprocessing techniques and noise reduction strategies are essential to mitigate these effects, improving training data quality and bolstering model performance. Additionally, the development of noise-robust architectures and training methods holds promise in enhancing model resilience to noise and variability in medical imaging data, ultimately augmenting their efficacy in clinical applications [13,14,15]. 

Regardless of the numerous techniques being used, accurate automatic segmentation in DXA imaging always remains a challenge [16,17,18,19,20,21,22]. Manual segmentation is time-consuming, requires an expert, and is impractical for analyzing large public datasets [16,18,19]. With the inherent limitations described in the references [23,24,25], the existing techniques are unsuitable for DXA image segmentation. All the limitations described in our previous work [26] forced us to new investigations, which led us to more precise segmentation with greater accuracy in DXA imaging. 

Integration of denoising techniques into convolutional neural network (CNN)-based DL models constitutes a critical step in the preprocessing pipeline. Before training, noisy DXA images undergo filtration using one or a blend of denoising methods to enhance their quality. These denoised images serve as input data for training the CNN model, enabling it to learn from cleaner and more representative image data. Subsequently, during inference, the trained CNN model is directly applied to noisy DXA images for femur segmentation, resulting in heightened accuracy and reliability in the segmentation process. To evaluate the impact of these denoising filters on DL-based image segmentation, we compiled the results by applying the CNN-based model to segment femur images and calculate BMD both with and without the application of denoising filters. This comparative analysis allows us to comprehensively understand the effectiveness of denoising techniques in enhancing the performance of DL-based segmentation algorithms. Preprocessing data (i.e., noise filters) and its effect on DXA image segmentation have not been fully investigated previously; in this study, we proposed preprocessing DXA images before they go to a DL modal.

We conducted comprehensive experiments to evaluate the effectiveness of the preprocessing steps and noise reduction effect over the recent DL-based approach for femur segmentation from DXA images. The best results were achieved with deeper fully convolutional neural network (FCNN)-based architecture with a Wavelet-based noise reduction filter than previously applied techniques. We obtained both high-energy (HE) and low-energy (LE) images from a DXA scan. To create images with a high contrast, both were merged. We investigated the impact of preprocessing filters on noise or imperfection removal in high-contrast image creation, and consequently its effect on DL-based segmentation and BMD analysis.

The main objective of this research is to find a solution that improves the image segmentation, BMD calculation, and consequent analysis of DXA image analysis. The rest of the paper is organized as follows. Section 2 explains the segmentation method of our model. Section 3 shows the proposed model results. Discussion about our method and results are presented in Section 4. Section 5 presents some concluding remarks. Finally, Section 6 presents some future work to be carried out. The main contribution of this research is highlighted as follows:

- Advancement in DXA Imaging Segmentation: Introduces a DL approach with enhanced image quality with various image denoising techniques for femur segmentation, improving osteoporosis diagnosis and bone mineral density calculation accuracy.
- Application of DL in Medical Imaging: Expands FCNN use in DXA imaging, showing potential for addressing segmentation challenges and improving diagnostic accuracy.
- Future Research Directions: Identifies areas for improvement in femur segmentation, stimulating further inquiry and innovation in medical imaging and DL applications.

## 2. Methods

Data from the femur were obtained through DXA scanning, resulting in high-energy (HE) and low energy (LE) images. Before high-contrast images were produced from the combination of HE and LE photos, various denoising techniques were used to remove imperfections from HE and LE images.

With its exceptional accuracy and efficiency in tasks ranging from organ segmentation to illness diagnosis, DL has completely changed the field of medical image analysis. Femur segmentation from DXA images is one such crucial task that helps with osteoporosis diagnosis and tracking. However, several variables, such as noise and image quality, can affect how effective DL models are in this area. To create robust and dependable segmentation algorithms, it is essential to comprehend how noise and image quality affect DL performance.

### 2.1. Data Preparation

We utilized 600 femur images obtained by a DXA scanner (OsteoPro MAX, Yozma BMTech Worldwide Co., Ltd., Seongnam-si, Gyeonggi-do, Republic of Korea). For model training and testing, and assessment of the accuracy of DL techniques, “ground truths” of manually segmented images and BMD results obtained by expert radiologists were collected for this study. Various denoising models were employed as a preprocessing step for high-contrast image generation and removal of imperfections from images. In the segmentation process, each pixel was categorized as either bone or soft tissue or air. For the five-fold evaluation test, the data were divided into two sets: 80% for training and 20% for independent testing. Subsequently, a test database was formed and fed into the proposed DL-based model lacking class labels, and the model generated output labels for each pixel of a test subject.

### 2.2. Image Generation

**Figure 1:**
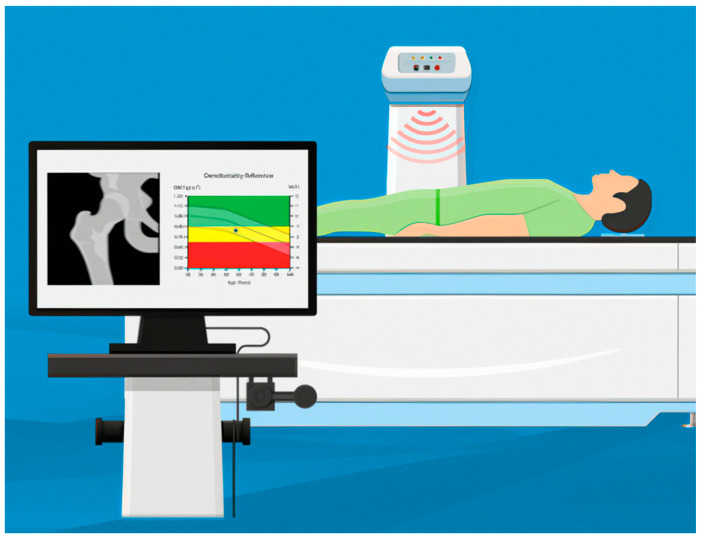
Acquire data with DXA machine scanning femur [30].

The domain of medical image analysis has witnessed recent advancements in DL techniques, which have demonstrated remarkable and state-of-the-art performance on vast datasets comprising numerous images across various categories. Nevertheless, these remarkable accomplishments underscore the susceptibility of different DL models to image quality concerns [27]. Thus, when considering DL models, image quality assumes a pivotal role. The enhancement of visual quality in images through contrast improvement and noise reduction can significantly impact image classification and segmentation [27,28,29]. In this investigative study, we acquire DXA data in the form of LE and HE images. These LE and HE images are merged to create a high-contrast display image, as depicted in Figure 1 [30]. Our study aims to investigate the influence of three distinct types of high-contrast display images generated by DXA scans on the outcomes of DL. These high-contrast display images encompass images of bone density (*IBD*), an image of HE, LE log ratio (*ILR*), and a collage image (*CI*). The IBD image, which serves as the initial output, can be generated by following these steps:

(1) $$IBD=\frac{R_{st}\times \frac{{\log HE}_{i}}{{HE}_{0}}-\frac{{\log LE}_{i}}{{LE}_{0}}}{u_{l}-u_{h}\times R_{st}},$$

(2) $$R_{st}=\frac{\log\frac{{LE}_{0}}{\frac{1}{n}\sum_{i=1}^{n}{LE}_{i}}}{\log\frac{{HE}_{0}}{\frac{1}{n}\sum_{i=1}^{n}{HE}_{i}}},$$

where *u_l_*, *u_h_*, are the constant values of a specific *LE* X-ray and *HE* X-ray, respectively. *HE*_0_ and *LE*_0_ are incident energy at the source X-ray, and *HEi*, *LEi* are detector counts at a specific scan location (i.e., image pixel). The BMD value at the bone region is always higher than the soft tissue region; therefore, from Equation (1), we get a brighter bone and darker soft tissue region image. 

The second image we generate from raw DXA data is *ILR*, which can be generated as follows:

(3) $$ILR=pow(T-\frac{\log\left({HE}_{i}+{LE}_{i}\right)}{\log\left(u_{l}+u_{h}\right)},B+C),$$

where *T* is a constant value and we used *T* = 2.0 as a constant value. Similarly, *B* and *C* represent image brightness and contrast enhancement. We used *B* and *C* as constant values to be determined from experiments. Using Equation (3), we get higher-contrast images with clear boundaries between bone, soft tissue, and air.

**Figure 2:**
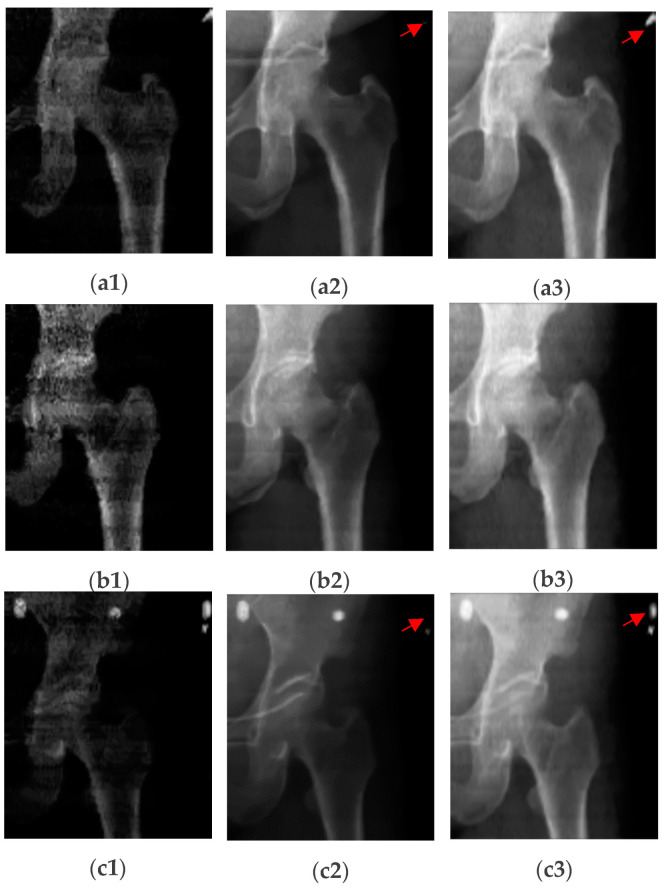
Generated high-contrast images from DXA scan. In this figure (**a1**,**b1**,**c1**) are IBD images, (**a2**,**b2**,**c2**) represent ILR images, and (**a3**,**b3**,**c3**) represent high contrast *CI* images. As indicated by the arrows, some information is hidden at the arrow positions in the ILR images, while this information is revealed in the *CI* images. We primarily use *CI* for the final segmentation of DXA images. These collage images are created by combining high-energy (HE) and low-energy (LE) images, enhancing the contrast and improving segmentation performance. Bone mineral density is calculated from the HE and LE images using different algorithms as in Equations (1) and (2).

The third image we generate from DXA data is *CI*, which can be formed as follows:

(4) $$CI=\left(\log\left(\frac{{HE}_{i}}{{LE}_{i}}\right)-\frac{\log\left(\frac{{LE}_{i}}{{HE}_{i}}\right)}{1+e^{-\delta \times \left(\log\left(\frac{{LE}_{i}}{{HE}_{i}}\right)-\mu \right)}}\right)\times \left(B+C\right)-log{LE}_{i},$$

where ‘δ’ is the standard deviation (STD) of the log (*LE*/*HE*) ratio and ‘μ’ is the mean of the log (*LE/HE*). The values of *B* and *C* represent image brightness and contrast values to be obtained from experiments. From Equation (4), we get a very high-contrast image with clear boundaries between different regions (i.e., bone, soft tissues, and air) compared to *IBD* and *ILR*. As we see from Figure 2, some information (most probably an artifact), indicated by red arrows in Figure 2(a2,a3), is hidden which appears in the *CI* and *IBD* images, as indicated by red arrows in Figure 2(c1,c3). So, *CI* gives a very high-contrast image with detailed DXA scan information. One drawback of the *ILR* image is that when an image contains an artifact, the image appears very black, as shown in Figure 2(c2). This problem does not affect *CI* images, as we see in Figure 2(c3). We normalize the intensities of the image from 0 to 255, and the final image is saved in ‘.png’ format to be used in the *DL* model. 

### 2.3. Noise in DXA Images

DXA images, while widely used for bone density assessment, are susceptible to various forms of noise. These noises can originate from factors such as equipment imperfections, patient movement during imaging, or interference from surrounding objects. Such noise can obscure important anatomical details and hinder accurate segmentation.

The presence of noise in DXA images can significantly affect the performance of DL models for femur segmentation. Noise introduces inconsistencies and irregularities in the image data, making it challenging for the model to distinguish between bone structures and background noise. As a result, DL algorithms may produce inaccurate segmentation, leading to erroneous clinical interpretations. Various preprocessing techniques can be employed to mitigate the adverse effects of noise on DL performance in femur segmentation. Additionally, data augmentation strategies such as random rotations, translations, and elastic deformations can help improve the robustness of DL models to noise.

Denoising images is a very important preprocessing step in image classification and segmentation [28,29]. S. Calderon et al. [28] studied the impact of denoising and contrast enhancement using a deceived non-local means (DNLM) filter in a CNN-based approach for age estimation using digital hand X-ray images. The results they obtained suggest that both image contrast enhancement and denoising can remarkably improve the results in a CNN-based model [28]. G. B. P. Costa et al. conducted a study about the effect of noise in image classification. Their study revealed that the image classification task was improved by the denoising process [29]. To improve the accuracy of image segmentation, noise reduction in both HE and LE images is an essential preprocessing procedure in DXA imaging. We employed and tested several denoising techniques, such as Non-local Mean Filter (NLMF), Gaussian filtering, and wavelet-based denoising to check the improvement in segmentation and BMD calculation using DL-based segmentation. The NLMF eliminates noise by comparing the similarity of patches through pixel neighborhoods. Gaussian filtering involves convolving the DXA image with a Gaussian kernel. Similarly, wavelet-based filtering in DXA imaging involves using wavelet transforms to analyze and process images. The denoising methods were configured with specific settings tailored to their respective algorithms. For instance, NLMF utilized optimal parameters such as a search window, a patch size, and a filtering strength (h), for effective noise reduction. Gaussian filtering involved setting parameters such as a standard deviation (sigma) and a kernel size. Wavelet-based denoising used a Daubechies (db2) wavelet, with a decomposition level, and applied thresholding parameter values.

#### 2.3.1. Non-Local Mean Filter 

The NLMF denoising technique is commonly used in medical imaging, including DXA imaging. In DXA imaging, where the images are often affected by noise due to factors such as low X-ray dose and scatter, denoising techniques like NLMF can be essential for improving image quality and enhancing the accuracy of subsequent analysis. Kim Kyuseok et al., (2020) [31] demonstrated through visual assessment and quantitative analysis that the NLM algorithm outperforms existing methods in processing casting images, offering an efficient solution to mitigating noise in high-energy X-ray imaging systems and potentially enhancing image restoration through accompanying software. Seungwan Lee et al., (2022) [32] introduce a newly improved non-local means (INLM) denoising algorithm tailored for X-ray images, accounting for the thickness of aluminum (Al) filters commonly used in X-ray systems, demonstrating its efficacy in reducing image noise. Results indicate that the proposed INLM algorithm, particularly when applied to X-ray images with a 5 mm Al filter thickness, exhibits superior performance in noise reduction and image evaluation compared to conventional methods, highlighting its importance for optimizing image processing applications in photon-counting X-ray systems.

The NLMF works by averaging the pixel values in an image, with the averaging process weighted based on the similarity between patches of pixels in the image. Unlike traditional filters that rely on local information, the NLM filter considers non-local similarities, meaning it compares patches of pixels from different image regions to determine the weighting for averaging. The approach of NLMF effectively removes noise while preserving image details, making it particularly suitable for medical imaging applications where preserving fine structures and details is crucial for accurate diagnosis and analysis. NLMF can help reduce the effects of noise, leading to clearer and more accurate DXA images, which in turn can improve the reliability of measurements such as BMD calculations and segmentation of bone structures.

**Figure 3:**
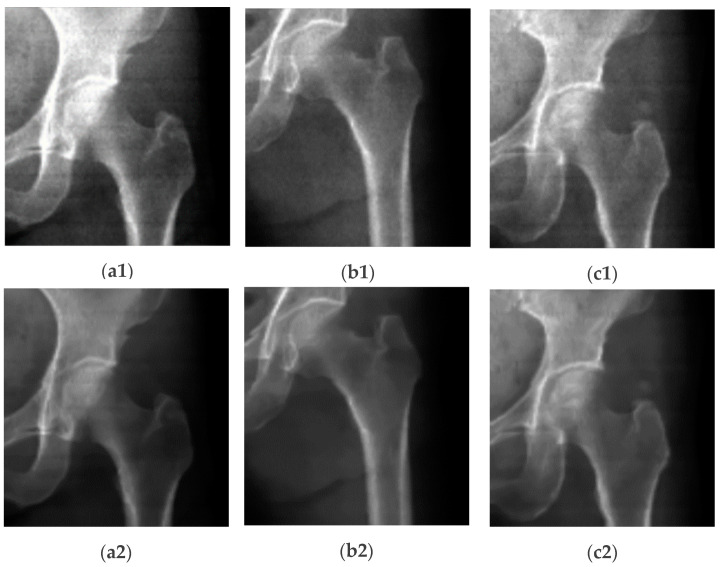
Denoising DXA images using NLMF. In this figure, (**a1**,**a2**,**a3**) represent three cases of high contrast *CI* images without using a denoising filter, while (**a2**,**b2**,**c2**) show the same images after applying NLMF denoising.

The optimized NLMF was tested and verified with the DXA images of the uniform femur phantom and real human femur images. Preliminary results showed that the signal-to-noise ratio (SNR) for high- and low-energy femur DXA images improved by 15.26% and 13.55%, respectively. Key parameters used include a search window size of 21 × 21 pixels, a patch size of 7 × 7 pixels, and a filtering strength (h) set to 10. Figure 3 shows some of the denoised results of femur DXA images using NLMF. More details about our NLMF work for denoising DXA images are available in references [33,34]. 

#### 2.3.2. Gaussian Filtering

Gaussian filtering (GF) is a common technique used for image processing and noise reduction. Gaussian filtering involves convolving the DXA image with a Gaussian kernel. This kernel is essentially a Gaussian distribution centered at the pixel of interest, with the values of neighboring pixels weighted according to their distance from the center. The Gaussian kernel is defined by its standard deviation, which determines the amount of smoothing applied to the image [35,36,37,38].

However, it is important to note that GF may also blur fine details in the image, so the choice of the filter’s parameters (such as the standard deviation of the Gaussian kernel) should be carefully considered based on the specific requirements of the application. The equation for GF in DXA image processing can be expressed as:

(5) $$G\left(i,j\right)=\frac{1}{2\pi \sigma^{2}}e^{-\frac{i^{2}+j^{2}}{2\sigma^{2}}\times I(i,j)}$$

where *G*(*i*,*j*) is the filtered image, *I*(*i*,*j*) is the original image, and *σ* is the standard deviation of the Gaussian distribution. This equation represents the convolution of the original image *I*(*i*,*j*) with a 2D Gaussian kernel. The Gaussian kernel is centered at (*i*,*j*) with values determined by the Gaussian function, where σ controls the width of the kernel and hence the amount of smoothing applied to the image. In our study, the Gaussian filter was implemented with a standard deviation (*σ*) of 1.5 and a kernel size of 5 × 5 pixels.

Nazia Fathima et al. (2020) proposed a novel approach for accurately measuring BMD from X-ray images using a modified U-Net with an attention unit [39]. The proposed method includes preprocessing steps, including Gaussian filtering, to enhance image quality. Results demonstrate improved segmentation accuracy and high classification accuracy for osteoporosis detection, validating the effectiveness of the approach.

#### 2.3.3. Wavelet-Based Methods

Wavelet-based denoising is often used in medical imaging. Wavelet-based denoising decomposes medical images into different frequency components using wavelet transforms. High-frequency noise in the decomposed image is then suppressed through thresholding methods, while preserving important diagnostic features. The denoised image is reconstructed, resulting in reduced noise and improved image quality suitable for clinical diagnosis and analysis [40,41]. G. Elaiyaraja et al., (2022) [42] proposed an optimal wavelet threshold-based denoising filter that effectively removes adaptive Gaussian noise from medical images, offering superior performance and efficiency. Farah Deeba et al., (2020) [43] proposed a wavelet-based Mini-grid Network Medical Image Super-Resolution (WMSR) method that enhances low-resolution medical images by leveraging the stationary wavelet transform (SWT). By combining wavelet sub-band images and utilizing sub-pixel layers, the model achieves superior performance in image reconstruction speed and quality.

Wavelet-based filtering in DXA imaging involves using wavelet transforms to analyze and process images [44,45]. Weiya Xie et al., (2021) [46] explored the application of photoacoustic time–frequency spectral analysis (PA-TFSA) for assessing bone mineral density (BMD) and structure. Utilizing wavelet transform-based PA-TFSA, simulations and experiments on bone samples revealed significant associations between frequency components and bone characteristics. Parameters derived from PA-TFSA, particularly mid band-fit and slope, demonstrated sensitivity in distinguishing between osteoporotic and normal bones. 

Wavelet transforms are powerful tools for image processing because they can represent both frequency and spatial information simultaneously. The wavelet transform decomposes an image into different frequency components. It consists of two main components: the scaling function (approximation) and the wavelet function (detail). The wavelet transform can be applied in either one dimension (1D) or two dimensions (2D). The continuous wavelet transform (CWT) of a signal or image *f*(*x*) is defined as

(6) $$W\left(a,b\right)=\int_{-\infty }^{\infty }f\left(x\right)\emptyset_{a,b}\left(x\right)dx$$

where $\emptyset_{a,b}\left(x\right)dx$ is the wavelet function, scaled by *a* and translated by *b*.

In practice, continuous wavelet transformation is often computationally expensive. Therefore, the discrete wavelet transform (DWT) is commonly used. It decomposes the image into different scales and orientations, resulting in a multi-resolution representation of the image. DWT is applied iteratively to decompose the image into approximation (low-frequency) and detail (high-frequency) coefficients. This can be represented mathematically as

(7) $$W\left(j,k\right)=\sum_{n}s_{j,k}\left(n\right).\emptyset (n-k)$$

where *W*(*j*,*k*) are the wavelet coefficients at scale *j* and position *k*, $s_{j,k}\left(n\right)$ are the scalling cofficents, and *Ø*(*n − k*) is the wavelet function.

Wavelet-based denoising involves thresholding the wavelet coefficients to remove noise while preserving image details. This is achieved by setting coefficients below a certain threshold to zero. Common thresholding methods include hard thresholding and soft thresholding.

Hard Thresholding (HT):

(8) $$T_{H}\left(x\right)=\left\{\begin{array}{c} x,\;\;if\;\left|x\right|>T\\ 0,\;\;otherwise \end{array}\right.$$

Soft Thresholding (ST):

(9) $$T_{S}\left(x\right)=sign\left(x\right).{\left(\left|x\right|-T\right)}_{+}$$

where *x* represents the wavelet coefficient, *T* is the threshold value, and (.)+ denotes the positive part of the function. If the absolute value of *x* is less than *T*, the coefficient is set to zero. This approach is straightforward and leads to sparse representations by eliminating coefficients that are considered noise.

Hard thresholding is advantageous in scenarios where the noise level is well-defined or known, as it can effectively suppress noise without overly distorting the image features. However, it can sometimes introduce artifacts in regions where the signal is weak but not strictly zero, leading to a loss of subtle details in the reconstructed image. In contrast to soft thresholding, which applies a smoother shrinkage to coefficients, hard thresholding offers a more aggressive noise reduction by eliminating coefficients below the threshold. Its effectiveness depends on selecting an appropriate threshold T, which balances noise suppression with the preservation of significant signal components. Using the Daubechies (db2) wavelet, with a decomposition level of 3, the soft thresholding achieved optimal denoising results.

### 2.4. Data Augmentation 

DL algorithms necessitate a substantial volume of data for effective training. However, the limited size of medical datasets presents a significant challenge in this regard. To meet the large dataset requirements of deep neural network training, the small data size is increased using data augmentation [47,48]. In this study, we addressed the data scarcity by randomly selecting 350 images and applying transformations such as image translations, and horizontal and vertical reflections, along with their subsequent scaling. These augmentations resulted in the expansion of our dataset to 1800 images, facilitating more robust training of deep neural networks.

### 2.5. Depp Learning Architecture

**Figure 4:**
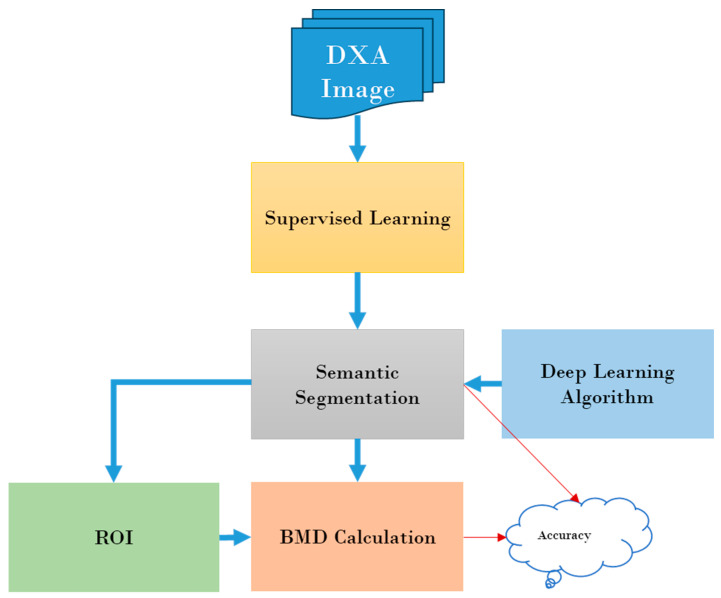
Overview of DXA image analysis using deep learning.

Figure 4 provides an overview of DXA image analysis employing DL methodologies. In this investigation, we introduce the U-Net, SegNet, and FCN approaches for femur segmentation from DXA images with preprocessing and postprocessing steps. The integration of the pre- and post-processing steps has been demonstrated to be crucial in significantly enhancing the efficacy of diverse tasks and applications. Through the utilization of pre-processing methodologies such as noise reduction, image standardization, and data enhancement, the original input data can be improved and optimized before its utilization in the model. These pre-processing approaches are employed to alleviate the impact of noise, variability, and discrepancies in the data to amplify the flexibility and precision of subsequent analyses or forecasts. Similarly, post-processing techniques like filtering, smoothing, and calibration can further perfect the outcomes generated by the model to ensure their alignment with specific quality or criteria. In essence, the incorporation of the pre- and post-processing stages plays a pivotal role in maximizing the efficiency and efficacy of the entire data processing pipeline to attain exceptional performance outcomes. 

FCNs are adept at generating dense predictions from input data of variable dimensions. Notably, both training and inference processes operate on the entire image concurrently through dense feedforward computation and backpropagation [49]. Unlike conventional CNN models featuring fully connected layers (FCL), FCNs exclusively consist of convolutional layers without any FCL, as indicated by their names. Within the suggested DL segmentation networks, we employ the sigmoid activation function in the activation layer to classify each pixel within the femur image into bone, soft tissue, or air categories. Further elaboration on the U-Net, SegNet, and FCN methodologies can be found in references [49,50,51]. The Adadelta optimization algorithm was employed to train all segmentation models, utilizing a batch size of 25. Initially, the learning rate was set to 0.2, with dynamic adjustments made throughout 200 epochs to optimize training. Weighted cross-entropy was utilized to compute the loss, aiming to minimize the overall loss *H* during the training phase as follows:

(10) $$H=-\sum_{c=1}^{M}y_{c}log({\hat{y}}_{c})$$

where y represents the ground truth labels and $\hat{y}$ represents the predicted map of segmentation. *M* represents the number of classes and ‘*c*’ represents classes (bone, tissue, air). The research was implemented in Python utilizing the Keras framework, with TensorFlow serving as the backend library.

### 2.6. Post-Processing

Unlike DL models, conventional semantic segmentation techniques typically necessitate the use of boundary smoothing filters to refine the femur bone boundaries delineated by the segmentation model and eliminate imperfections. In a prior investigation, we employed a binary smoothing filter to eradicate inaccuracies from segmented DXA images [23,24]. Addressing imperfections introduced during pixel labeling in image segmentation using machine learning is challenging. Morphological image processing (MIP) effectively handles these issues by considering the image’s shape and structure. In instances where sometimes DL labeling of femur boundaries results in non-smooth contours, binary smoothing proves effective in refining femur object boundaries by eliminating small-scale noise while preserving large-scale features. For additional information regarding binary smoothing, please refer to our previous work cited in [23,24].

### 2.7. Evaluation and Performance Analysis

To evaluate the performance of DL-based predictions with preprocessing noise filters compared to ground truth using a range of evaluation metrics, these metrics—including True Positives (TP), True Negatives (TN), False Positives (FP), and False Negatives (FN)—are recorded for each segmented pixel in the DXA image to quantify the accuracy of the models over the total number of pixels (n = TP + TN + FP + FN) in an image. These metrics were calculated over the entire dataset of test images.

One of the key evaluation metrics used in the study was Intersection over Union (IOU), which is a measure commonly used to assess the accuracy of segmented objects compared to ground truth. IOU is calculated as the ratio of the area of intersection between the segmented object and ground truth to the area of their union. This metric indicates how well the segmented object aligns with the ground truth, with higher IOU values indicating better alignment.

Additionally, Sensitivity (True Positive Rate, TPR) and Specificity (True Negative Rate, TNR) were used to measure the proportion of positive and negative pixels accurately identified, respectively. Sensitivity measures the proportion of bone tissue pixels correctly identified in the femur DXA image, while Specificity measures the proportion of soft tissue pixels correctly identified. The False Positive Rate (FPR) and False Negative Rate (FNR) were also calculated to quantify the rate of misclassification of positive and negative pixels, respectively.

We employed a threshold-based approach to assess the overall accuracy of the segmentation methods. A segmentation method was considered successful if IOU was >0.92, Sensitivity was >95%, or Specificity was ≥93%. These thresholds were chosen to ensure that the segmentation methods accurately identified both bone and soft tissue regions in the DXA images.

We used a five-fold cross-validation approach, by dividing the dataset as 80% for training and the remaining 20% for independent testing. Test data were swapped with training data during cross-validation to ensure a robust evaluation of the segmentation methods. Finally, the segmentation methods’ retrieved ground truths (RGT) and manual ground truths (GT) were applied to the test data in each cross-validation fold to compare their performance. For more details, visit our previous work referenced in [23,24,26], as the same evaluation and performance analysis strategy was adopted to evaluate the accuracy of this work.

(11) $$Accuracy=100\times \frac{1}{N}\sum_{i=1}^{N}x_{i}\;where,\;{f(x}_{i})=\left\{\begin{array}{l} 0,\;\;\;\;if\;JI<0.92\;||\varepsilon <95\;||\vartheta <93\\ 1,\;\;\;\;else \end{array}\right.,\;JI=\frac{\left|A_{c}\cap B_{c}\right|}{{|A}_{c}|+{|B}_{c}|}.$$

where *JI* is the Jaccard index or IOU, epsilon (ε) is the image segmentation sensitivity, and *ϑ* is the image segmentation specificity. |*A_c_*| is the set of pixels predicted as class c and |*B_c_*| is the set of pixels in the ground truth labeled as class *c* (i.e., bone, soft tissue, or air). ∣*A_c_*∩*B_c_*∣ is the number of pixels common to both sets (true positives). 

Furthermore, we also calculated the average Dice score (*DISC*) for image segmentation involving three regions (bone, soft tissue, and air). We compute the Dice score separately for each class and then average them. The *DISC* was calculated using the following formula:

(12) $$DISC=2\times \frac{\left|A_{c}\cap B_{c}\right|}{{|A}_{c}|+{|B}_{c}|},$$

We calculated the mean Dice score by averaging the Dice scores for all classes:

(13) $${DISC}_{{Mean}_{i}}=\frac{1}{N}\sum_{c=1}^{N}D_{c},$$

where *N* is the number of classes (in this case, *N* = 3). Finally, we calculated the average *DISC* for the overall test dataset.

## 3. Results

### 3.1. Data

We used the same dataset used in our previous studies [23,24], with some additional femur images acquired on a DXA scanner (OsteoPro MAX, Yozma BMTech Worldwide Co., Ltd., Seongnam-si, Gyeonggi-do, Republic of Korea). Radiology experts manually segmented femur images as the “ground truth”. We extracted manually annotated images from the DXA system in the “.png” format, alongside high-contrast images, to train and test our DL models. Each and every pixel in the femur image was annotated and assigned a class label (i.e., either bone, soft tissue, or air). 

### 3.2. Noise Reduction

**Figure 5:**
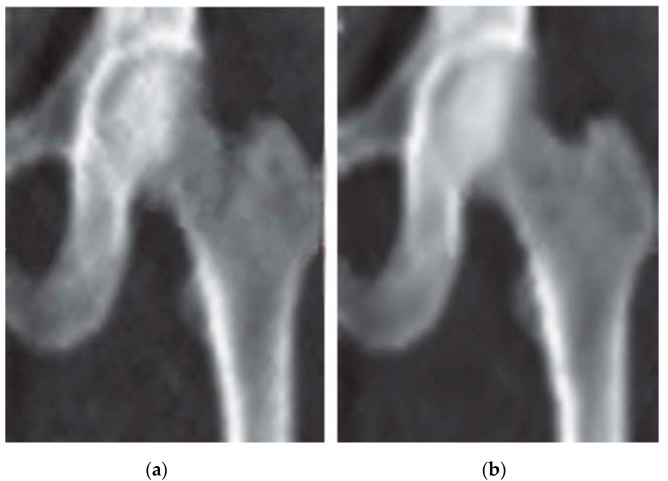
Image denoised with NLMF: (**a**) original and (**b**) denoised image.

The proposed denoising techniques via NLMF, Gaussian filtering, and wavelet-based methods for DXA images were evaluated experimentally with femur data. The performances of denoising filters was evaluated quantitatively in terms of mean-to-standard deviation ratio (MSR), signal-to-noise ratio (SNR), and contrast-to-noise ratio (CNR). The proposed noise reduction techniques significantly improve the quality of DXA images and image segmentation results, while preserving the fine details of anatomical structures. Compared to other denoising methods, wavelet-based filtering has shown higher performance in the case of the average improvement ratio of MSR, SNR, and CNR and improves the quality of DXA images. Table 1 presents the effects of NLMF, GF, and CWT filters. MSR, or mean-to-standard deviation ratio, quantifies the quality of DXA images by comparing the mean pixel value across the entire image to the standard deviation within a specified region of interest (ROI) of 10 × 10 pixels, as outlined in [34,38,52]. A higher MSR value indicates superior image quality, reflecting clearer and more defined anatomical details. SNR, measured in decibels, was calculated for both original and denoised DXA images. It evaluates the signal strength relative to the noise level, with μo representing the mean pixel value of the bone object and σROIbg denoting the standard deviation in a background ROI of equivalent size. Higher SNR values indicate effective noise reduction and improved image clarity. Similarly, CNR in decibels was estimated to assess the contrast between the bone and background regions, providing insights into the image’s diagnostic utility and overall enhancement achieved through denoising techniques. A sample of original and denoised images with NLMF is shown in Figure 5. 

Table 1 illustrates the effectiveness of various denoising techniques applied to femur DXA images. The metrics MSR, SNR, and CNR are critical for evaluating image quality and noise reduction. The results indicate that wavelet-based methods, particularly Continuous Wavelet Transform—Soft Thresholding (CWT-ST), provide the best performance in enhancing image quality and reducing noise, followed by Continuous Wavelet Transform—Hard Thresholding (CWT-HT), NLMF, and GF. These improvements are essential for accurate segmentation and reliable BMD calculations.

### 3.3. Segmentation

**Figure 6:**
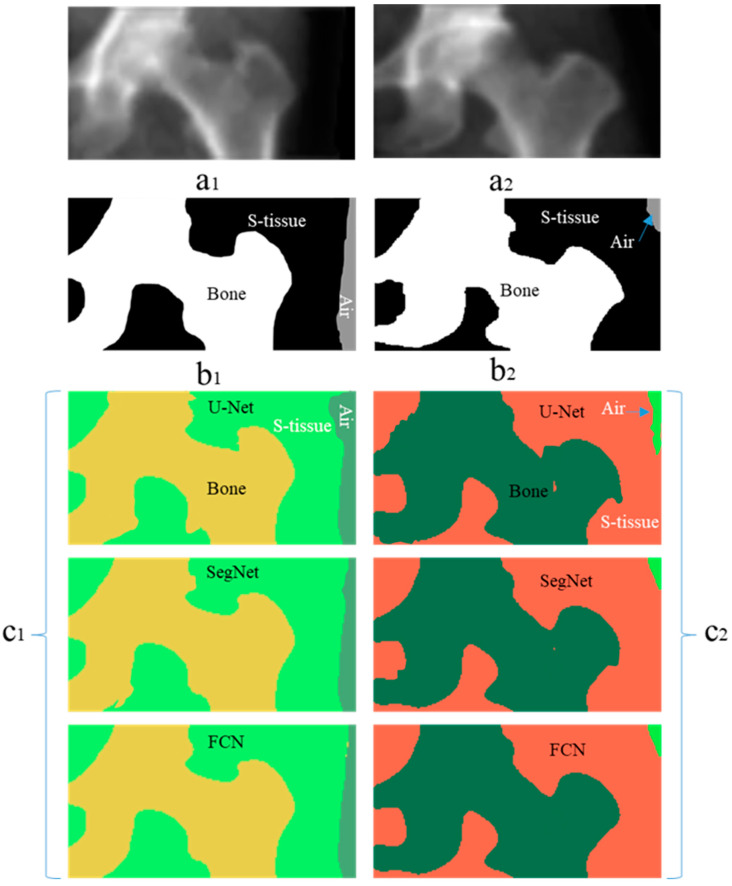
Selected results of two cases segmented with SegNet, U-Net, and FCN without smoothing filters. In this figure, (**a1**,**a2**) are the original images, while (**b1**,**b2**) represent the ground truths. (**c1**) on left side and (**c2**) on right side depict the segmentation masks of the original images (**a1**,**a2**) segmented using various DL algorithms without any post-processing applied. These two images were acquired using two distinct DXA imaging devices. The blue arrows indicate air labels in the images.

**Figure 7:**
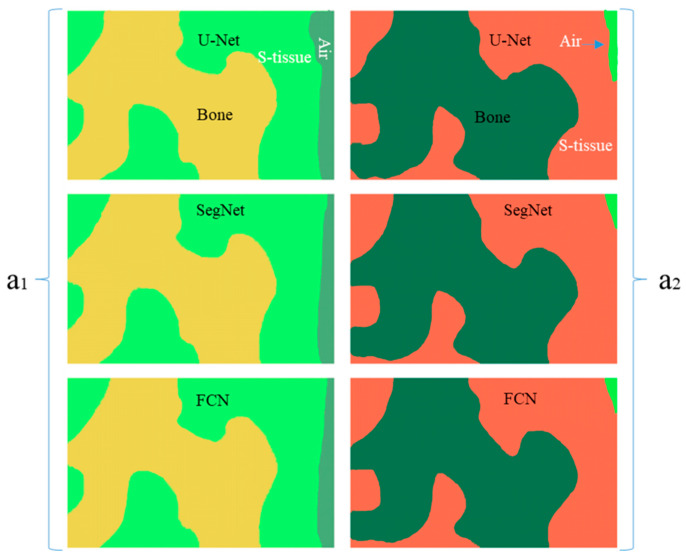
A binary smoothing filter was applied to the DL output in Figure 6. The results depict two cases segmented using SegNet, U-Net, and FCN with the addition of a binary smoothing filter as post-processing. In this figure, (**a1**) on the left side shows the segmentation mask corresponding to a1 in Figure 6, while (**a2**) on the right side displays the segmentation mask corresponding to (**a2**) in Figure 6. These masks were generated using various DL algorithms (U-Net, SegNet, and FCN, each labeled accordingly) and then smoothed with a binary smoothing filter. The blue arrows indicate air labels in the images.

This section presents the performance of U-Net, SegNet, and FCN approaches using test data (i.e., 250 femur images). Some of the selected results from U-Net, SegNet, and FCN output with non-smooth contours, and binary smoothed contours are shown in Figure 6 and Figure 7, respectively. Figure 6 presents the raw output of DL models without any additional post-processing applied. This means that the segmentation results shown in Figure 6 are directly generated by the DL algorithms (SegNet, U-Net, and FCN) without any further enhancement or adjustment.

In contrast, Figure 7 illustrates the results of the same DL models after applying a binary smoothing filter as post-processing. The binary smoothing filter is applied to the segmentation masks obtained from the DL models to refine and smooth out the boundaries and contours of the segmented regions. This post-processing step aims to improve the visual clarity and accuracy of the segmentation results by reducing pixel-level noise and inconsistencies in the DL outputs.

**Figure 8:**
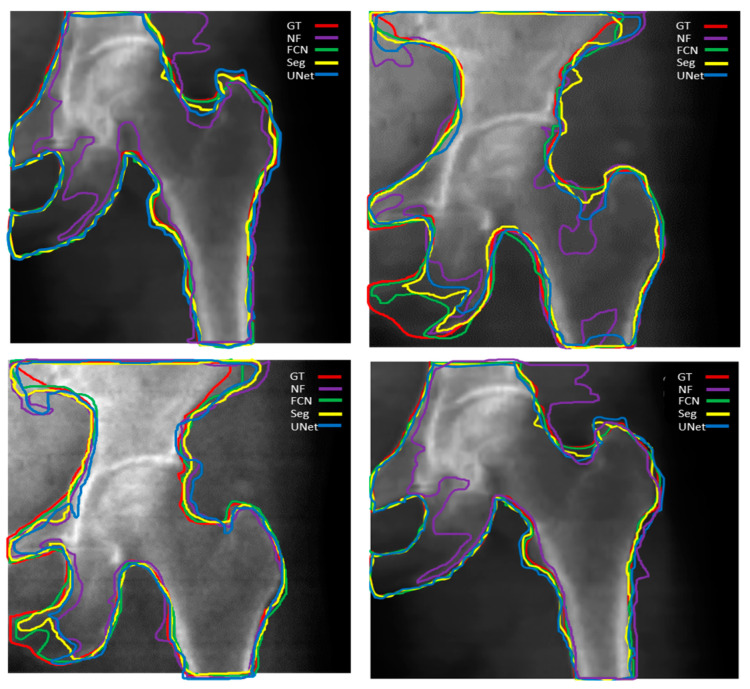
Predicted femur boundaries using SegNet, U-Net, and FCN segmentation models. Ground truth femur boundaries are outlined in red, while the predicted boundaries are represented by yellow (SegNet), blue (U-Net), green (FCN), and purple (FCN with no filter). The above segmentation results were obtained following a preprocessing with NLMF.

Thus, Figure 7 provides a comparison to Figure 6 by demonstrating how the application of a binary smoothing filter can potentially enhance the segmentation outcomes produced by the DL models, particularly in terms of achieving smoother and more coherent boundaries for the identified regions of interest. Table 2 shows the segmentation performance results in terms of average accuracy computed using the JI, sensitivity, and specificity of all test images. In addition, the average Dice score was calculated for bone, soft tissue, and air region in each image, and finally, a combined average Dice score for overall test data of each segmentation model was calculated. A couple of the predicted segmentation contours by different segmentation models using NLMF are shown in Figure 8. 

Table 2 highlights the segmentation performance of three deep learning models—SegNet, U-Net, and FCN—under various pre-processing conditions applied to femur DXA images. The table reveals that the FCN, particularly when combined with CWT-ST and a binary smoothing filter, achieves the highest segmentation accuracy (98.84%) and DISC (96.68%), significantly outperforming the other models. This suggests that the FCN’s architecture, coupled with effective denoising through wavelet-based soft thresholding, is particularly well-suited for this task. Moreover, the table underscores the positive impact of pre-processing techniques, with all models showing improved performance when any form of denoising is applied. Among these techniques, CWT-ST and CWT-HT consistently deliver superior results compared to NLMF and Gaussian filtering. This performance boost can be attributed to the wavelet methods’ ability to preserve fine details while effectively reducing noise. 

Our evaluation of 250 test femur images using DL models with a noise reduction pre-processing step demonstrated superior performance compared to simple DL methods, particularly in high-contrast femur sections (femur head and shaft) and challenging areas (greater and smaller trochanteric and ischium). The data were collected on multiple devices, and models covered the diversity well and performed competently. These findings emphasize the importance of selecting both advanced pre-processing strategies and robust model architectures to achieve optimal segmentation accuracy in femur DXA imaging.

### 3.4. BMD Analysis

We conducted a comparison of BMD between manually segmented images and those segmented by our models. Initially, a set of 100 femur images was randomly chosen and given to three individuals to manually segment the femur, select regions of interest (ROIs), and calculate BMD at three distinct regions: the femur neck, ward, and greater trochanter (G.T.). Subsequently, the average BMD value was computed from the three expert readings for each region, and these estimations were then compared to those obtained through model-based segmentation to assess consistency. Finally, we conducted a statistical analysis, calculating the correlation (R^2^) to evaluate the correlation of BMD measurements between different segmentation methods and manual segmentation. The FCN segmentation method with a CWT as preprocessing and binary smoothing of object boundaries as a post-processing step exhibited the highest correlation, as indicated in Table 3.

The findings underscore the superior “sensitivity, specificity, and accuracy” of the FCN method with preprocessing and postprocessing steps compared to other models, providing a robust solution for segmentation challenges in DXA imaging. While CNN is acknowledged as an innovative segmentation method, its reliance on large training datasets poses a limitation. To address this, we implemented transfer learning to enhance the training efficiency of DL models using small femur DXA images. Leveraging the weights of the pre-trained VGG-16 network from the extensive ImageNet dataset, our study on femur segmentation demonstrates that current DL models outperform previously utilized methods [23,24,25,26]. 

Training CNN-based networks with limited data poses a significant challenge in DL. Without optimized techniques and data augmentation, achieving satisfactory performance can be daunting. Therefore, employing appropriate optimization methods, data augmentation strategies, and transfer learning can greatly aid in training a reliable segmentation network. Transfer learning involves fine-tuning a deep network that has been pre-trained on either medical images or general datasets. When combined with data augmentation, transfer learning offers an additional distinct solution with numerous parameters. To tackle this challenge effectively, we successfully implemented transfer learning in our DXA image analysis, leveraging pre-trained models from ImageNet to enhance performance. 

Table 3 presents the R^2^ between BMD measurements obtained from manual segmentation and various DL models across different denoising methods. This correlation was assessed at three distinct regions of the femur: the neck, ward, and greater trochanter (G.T.). The results indicate that the FCN model, particularly when using Continuous CWT-ST as a preprocessing method and binary smoothing as a postprocessing step, achieved the highest overall mean correlation (R^2^ = 0.9928). This high correlation suggests that the FCN model with CWT-ST preprocessing and binary smoothing most accurately replicates expert manual segmentation.

In comparison, other models and denoising techniques showed varied performance. For instance, SegNet and U-Net with NLMF also demonstrated strong correlations (mean R^2^ of 0.9736 and 0.9755, respectively), but not as high as the FCN with CWT-ST. Models without any noise filtering consistently exhibited lower correlations, highlighting the importance of denoising in improving segmentation accuracy. Further investigations may discover the rational serviceability of the FCN and other DL models in the clinical diagnosis of osteoporosis and the prediction of fracture risk. All DL-based models have proven to have better performance than previously applied techniques. The study has shown that convolutional networks can effectively be utilized with high performance on a small clinical data set using transfer learning in semantic segmentation.

## 4. Discussion

The study investigated the influence of noise and image quality on the performance of DL models for femur segmentation from DXA images. A dataset comprising DXA images with varying levels of noise and image quality was utilized to assess the effectiveness of different denoising techniques in improving segmentation accuracy.

Firstly, the impact of noise on segmentation performance was evaluated. DXA images with simulated Gaussian noise at different levels were subjected to segmentation using a baseline CNN model. Results demonstrated a noticeable decrease in segmentation accuracy with increasing noise levels. However, when denoising techniques such as Gaussian filtering, CWT filtering, and Non-local Mean Filter were applied as preprocessing steps, the segmentation accuracy improved significantly across all noise levels. Particularly, the Wavelet-based Mean Filter exhibited the highest efficacy in reducing noise and enhancing segmentation accuracy.

Secondly, the influence of image quality on segmentation performance was analyzed. DXA images with varying levels of blur and contrast were utilized to assess the robustness of the CNN model to image quality variations. It was observed that images with low contrast and blur presented challenges for the CNN model, leading to decreased segmentation accuracy. Nevertheless, by applying preprocessing techniques such as contrast enhancement and wavelet-based filtering, the model demonstrated improved performance in segmenting femur structures from low-quality images.

Furthermore, the combined effect of noise and image quality on segmentation performance was investigated. DXA images with simultaneous variations in noise and image quality were used to simulate real-world scenarios. The results indicated that denoising techniques played a crucial role in mitigating the adverse effects of both noise and low image quality on segmentation accuracy. Wavelet-based methods, in particular, exhibited robust performance in preserving image details while reducing noise, resulting in improved segmentation accuracy even in challenging imaging conditions.

Image denoising plays a crucial role in enhancing the performance of CNN-based DL models, particularly in medical image analysis tasks such as femur segmentation from DXA images. Various denoising techniques, including Non-local Mean Filter, Gaussian filtering, median filtering, and wavelet-based methods, contribute to improving the robustness and accuracy of CNN models by reducing the impact of noise on image data.

Incorporating FCN-based segmentation with a wavelet-based preprocessing filter, and a binary smoothing filter as a postprocessing step, we observed a significant enhancement in femur segmentation accuracy and BMD calculation in DXA images. Our findings demonstrated that the FCN, when coupled with a wavelet-based filter, outperformed alternative segmentation techniques, achieving a notable accuracy rate. The application of wavelet-based filtering as a preprocessing step effectively reduced noise and enhanced image quality, thereby facilitating more precise femur segmentation in DXA imaging. Furthermore, the combination of FCN and awavelet-based filter exhibited robust performance across a range of imaging conditions, including challenging regions like the greater and smaller trochanteric areas, as well as high-contrast regions such as the femur head and shaft. These results underscore the potential of FCN-based segmentation with wavelet-based filtering as a reliable approach for accurate femur segmentation in DXA imaging, offering superior sensitivity, specificity, and overall accuracy compared to traditional methods.

The results highlight the significance of incorporating denoising techniques into CNN-based DL models for femur segmentation from DXA images. By effectively mitigating the influence of noise and image quality variations, these techniques enhance the accuracy and reliability of segmentation, facilitating more precise diagnosis and monitoring of osteoporosis.

## 5. Conclusions

Segmentation of the femur in DXA images poses challenges due to factors like reduced contrast, noise, and variations in bone shape. This study investigated the impact of noise on DL techniques for femur segmentation, incorporating noise reduction techniques to enhance DL-based models’ accuracy. By applying CNN to DXA images with and without noise reduction filters, we observed that the FCNN outperformed the use of noise reduction algorithms before model training, resulting in precise bone density calculation and improved osteoporosis diagnosis. The study demonstrated a higher accuracy of 98.84% for different segmentation methods and a significantly higher correlation (R2 = 0.9928) for BMD measurement compared to manual segmentation. This research contributes to advancing DXA imaging segmentation, enhancing diagnostic accuracy, and stimulating further inquiry into medical imaging and DL applications. The incorporation of Non-local Mean Filter, Gaussian filtering, and wavelet-based methods for image denoising in CNN-based DL models significantly contributes to improving the performance and robustness of femur segmentation from DXA images, ultimately enhancing the quality of osteoporosis diagnosis and patient care.

## 6. Future Directions

In the realm of medical imaging, future directions for femur segmentation from DXA images encompass several promising avenues. One avenue involves delving into advanced denoising techniques, such as deep learning-based models or adaptive algorithms, to further refine noise reduction strategies and enhance the accuracy of segmentation. Additionally, exploring the fusion of DXA images with other modalities like MRI or CT scans could unlock synergistic benefits, improving segmentation accuracy by leveraging complementary information from diverse imaging sources. Transfer learning and domain adaptation techniques offer another promising pathway, enabling model training on diverse datasets and enhancing generalization to different imaging conditions and patient populations. Moreover, developing methods for uncertainty quantification could provide valuable insights into the reliability of segmentation results, facilitating more informed decision-making in clinical settings. Clinical validation studies are essential for assessing the real-world performance and clinical utility of DL-based segmentation models, necessitating collaboration with medical professionals and institutions for rigorous validation against ground truth annotations and clinical outcomes. User-friendly software tools and platforms for DXA image segmentation should be developed to streamline integration into existing medical workflows and promote widespread adoption. Longitudinal studies exploring the prognostic value of segmentation-based biomarkers for predicting clinical outcomes, such as fracture risk or treatment response, offer valuable insights into personalized risk assessment and treatment planning in osteoporosis management. Lastly, addressing ethical considerations and potential biases in DXA image segmentation is paramount for ensuring equitable and unbiased healthcare delivery, requiring robust strategies for bias mitigation, fairness assessment, and transparency in model development and deployment.

## Figures and Tables

**Table 1:** Denoising technique performance analysis for femur DXA images. The results are presented in this table as mean values from all evaluated images.

Technique	Index	Image	
		Original	Denoised
NLMF	MSR	73.31	77.01
	SNR	5.26	5.74
	CNR	27.54	31.11
GF	MSR	73.31	74.15
	SNR	5.26	5.54
	CNR	27.54	29.26
CWT-ST	MSR	73.31	**78.21**
	SNR	5.26	**6.27**
	CNR	27.54	**32.63**
CWT-HT	MSR	73.31	77.81
	SNR	5.26	6.25
	CNR	27.54	31.41

**Table 2:** Segmentation performance of different methods on the test dataset.

DL Model	Trainable Parameters	Loss Function	Optimization Algorithm	Pre-Processing (Denoising Method)	Segmentation Accuracy (%)	DISC (%)
SegNet	13.3M	Cross-Entropy	Adam	No pre-processing	88.12	75.24
				NLMF	92.99	84.98
				GF	92.35	83.70
				CWT-ST	96.63	92.26
				CWT-HT	93.68	86.36
U-Net	12.7M	Dice Loss	Adam	No pre-processing	86.32	71.64
				NLMF	91.56	82.12
				GF	89.98	78.96
				CWT-ST	94.14	87.28
				CWT-HT	91.89	82.78
FCN	134.5M	Cross-Entropy	Adam	No pre-processing	89.36	77.72
				NLMF	94.76	88.52
				GF	93.89	86.78
				CWT-ST	**98.84**	**96.68**
				CWT-HT	94.97	88.94

**Table 3:** Correlation record of BMD measurements between different segmentation methods to manual segmentation.

DL Model	Denoising Method	R^2^			
		Neck	Ward	G.T.	Mean
SegNet	Without noise filter	0.9029	0.9108	0.9192	0.9109
	NLMF	0.9819	0.9798	0.9592	0.9736
	GF	0.9231	0.9307	0.9382	0.9306
	CWT-ST	0.9331	0.9467	0.9481	0.9426
	CWT-HT	0.9288	0.9348	0.9421	0.9352
U-Net	Without noise filter	0.8929	0.9018	0.8892	0.8946
	NLMF	0.9811	0.9896	0.9560	0.9755
	GF	0.8989	0.9102	0.9013	0.9034
	CWT-ST	0.9601	0.9634	0.9418	0.9551
	CWT-HT	0.9599	0.9595	0.9388	0.9527
FCN	Without noise filter	0.9413	0.9349	0.9324	0.9362
	NLMF	0.9698	0.9789	0.9814	0.9767
	GF	0.9479	0.9398	0.9404	0.9427
	**CWT-ST**	**0.9913**	**0.9949**	**0.9924**	**0.9928**
	CWT-HT	0.9734	0.9829	0.9816	0.9795

## Data Availability

No new data were created or analyzed in this study.
